# Assessing the risk of angiotensin receptor blockers on major cardiovascular events: a systematic review and meta-analysis of randomized controlled trials

**DOI:** 10.1186/s12872-020-01466-5

**Published:** 2020-04-21

**Authors:** Yara Wanas, Rim Bashir, Nazmul Islam, Luis Furuya-Kanamori

**Affiliations:** 1 Department of Population Medicine, College of Medicine, QU Health, Qatar University, Doha, Qatar; 2 Department of Public Health, College of Health Sciences, QU Health, Qatar University, Doha, Qatar; 3 Research School of Population Health, ANU College of Health and Medicine, Australian National University, Acton, Australia

**Keywords:** Cardiovascular events, Angiotensin receptor blockers, Meta-analysis, Risk

## Abstract

**Background:**

Angiotensin receptor blockers (ARBs) are commonly used as a treatment for many cardiovascular diseases, but their safety has been called into question. The VALUE trial found an increased risk of myocardial infarction in participants receiving ARBs compared to other antihypertensive. The aim of the meta-analysis was to synthetize the available evidence of randomised controlled trials (RCTs) and elucidate if ARBs increase the risk of cardiovascular events.

**Methods:**

A comprehensive search was conducted to identify RCTs that assessed the safety of ARBs. Titles and abstracts of all papers were independently screened by two authors. Data extraction and quality assessment were also performed independently. The relative risk (RR) of all-cause mortality, myocardial infarction, and stroke were pooled using the IVhet model. Multiple sensitivity analyses were conducted to assess the effect of ARBs by restricting the analysis to different participants’ characteristics.

**Results:**

Forty-five RCTs comprising of 170,794 participants were included in the analysis. The pooled estimates revealed that ARBs do not increase the risk of all-cause mortality (RR 1.00; 95%CI 0.97–1.04), myocardial infarction (RR 1.01; 95%CI 0.96–1.06), and stroke (RR 0.92; 95%CI 0.83–1.01). The sensitivity analysis did not yield a particular group of patients at increased risk of cardiovascular events with ARBs. Risk of all-cause mortality and stroke decreased with ARB when the proportion of smokers in a population was < 25% (RR 0.91; 95%CI 0.84–0.98) and in females (RR 0.76; 95%CI 0.68–0.84), respectively.

**Conclusions:**

*A*RBs do not increase the risk of major cardiovascular events and are safe for use in patients.

## Background

Cardiovascular diseases (CVDs) remain one of the most prevalent non-communicable diseases and impose a great burden on the healthcare systems. Globally, an estimated 16.7 million deaths in the year 2010 were attributed to CVD with projections showing a staggering 23.3 million deaths by 2030 [1]. Hypertension is the leading risk factor for CVD and it is associated with 57 million disability adjusted life years (DALYs) worldwide [2].

It is well known that the risk of major cardiovascular events can be reduced by a wide spectrum of antihypertensive drugs including angiotensin receptor blockers (ARBs) [3]. This type of drug works by inhibiting the angiotensin II receptors, thus causing systemic vasodilatation, thereby aiding in the reduction of blood pressure [4]. ARBs are one of the most common drugs used for controlling blood pressure, treating heart failure, and preventing kidney failure in people with diabetes or hypertension [5]. However, the safety of ARBs in comparison to other anti-hypertensive medications has been called into question.

The VALUE trial found that ARBs (valsartan) increased the risk of myocardial infarction (fatal and non-fatal) by 19% compared with calcium channel blockers (amlodipine) [6]. This observation led many researchers to examine cautiously the evidence surrounding ARBs and myocardial infarction. For example, the point estimate of the CHARM-alternative trial suggests a 36% increase in myocardial infarction with candesartan (versus placebo) regardless of the reduction in blood pressure [7]. On the other hand, the TRANSCEND trial found an 8% decrease in risk of cardiovascular admissions for those on telmisartan compared to placebo [8].

Angiotensin-converting-enzyme (ACE) inhibitors are known to have a cardioprotective effect and the safety profile of ACE inhibitors have been shown not to differ from ARBs [9]. Hence it was unclear the mechanism that could explain an increase in risk of myocardial infarction with ARBs. Due to the wide use of ARBs for many CVDs and the contradictory results, we decided to conduct a systematic review and meta-analysis of randomised controlled trials (RCTs) to elucidate the cardiovascular safety profile of ARBs.

## Methods

Findings of this systematic review and meta-analysis are presented according to PRISMA reporting guidelines [10].

### Search strategy and selection criteria

A systematic search was conducted in PubMed in September 2018. The following search terms were included: randomized controlled trial, angiotensin receptor antagonist, cardiovascular disease, and mortality. The full search strategy is shown in the supplementary material (S1). To achieve a comprehensive evaluation of the published evidence, the systematic search was supplemented with a similarity search (i.e. the first 20 related citations of each included paper) as well as hand search of the reference lists of relevant studies. Titles and abstracts were uploaded on Rayyan (http://rayyan.qcri.org/) [11] for the screening process. Two authors (YW and RB) independently screened all the records by title and abstract. Disagreements were resolved through author consensus and involvement of a third author (LFK).

The inclusion of studies was restricted to human studies; RCTs comparing ARBs versus a control (either a placebo or another antihypertensive medication); follow-up of at least 12 months; and reported all-cause mortality, myocardial infarction, and stroke as outcomes. Recurrent myocardial infarction and stroke were also considered if the study only included patients that have had recently experienced myocardial infarction or stroke. Observational studies, studies where ARBs were not the first line of treatment, and conference abstracts were excluded.

### Data extraction and quality assessment

The number of participants and the number events (i.e. all-cause mortality, myocardial infarction, and stroke) in each intervention group (ARBs [active] and non-ARBs [control]) were extracted. In addition, study characteristics (e.g. study sites and follow-up period) and participants’ characteristics (e.g. mean age, proportion of males, mean BMI) were extracted. The Cochrane Collaboration’s tool for assessing risk of bias in randomized trials [12] was used to assess the risk of bias of the included studies.

### Statistical analysis

The outcomes of interest were the relative risks (RRs) of all-cause mortality, myocardial infarction, and stroke with ARBs compared to the control group. The inverse variance heterogeneity (IVhet) model was used to pool the effect size [13]. The *I*^*2*^ index was used to assess heterogeneity among studies, an *I*^*2*^ > 50% was considered significant heterogeneity.

Sensitivity analyses were conducted to identify potential scenarios where ARBs increase the risk of all-cause mortality, myocardial infarction, and stroke. The following analyses restricting the meta-analysis to: control group (active medication, only ACE inhibitors, or placebo); follow-up period (≤40 weeks or > 40 weeks); proportion of males (≤50% or > 50%); age (≤65 years or > 65 years); BMI (normal range or overweight/obese); elevated total cholesterol (≥200 mg/dL); elevated LDL (≥120 mg/dL); decreased HDL (< 50 mg/dL); elevated triglyceride (≥150 mg/dL); proportion of smokers (< 25% or ≥ 25%); only patients with hypertension; only patients with or without chronic heart failure; only patients with or without diabetes mellitus; only patients with ischemic/coronary artery disease; and only patients with chronic kidney disease.

Publication bias was assessed through visual inspection of funnel and Doi plots and statistically through the Egger’s regression *p*-value and the LFK index [14]. All the analyses were conducted in Stata MP 14 (StataCorp, College Station, TX, USA).

## Result

### Study selection and study characteristics

**Fig. 1:**
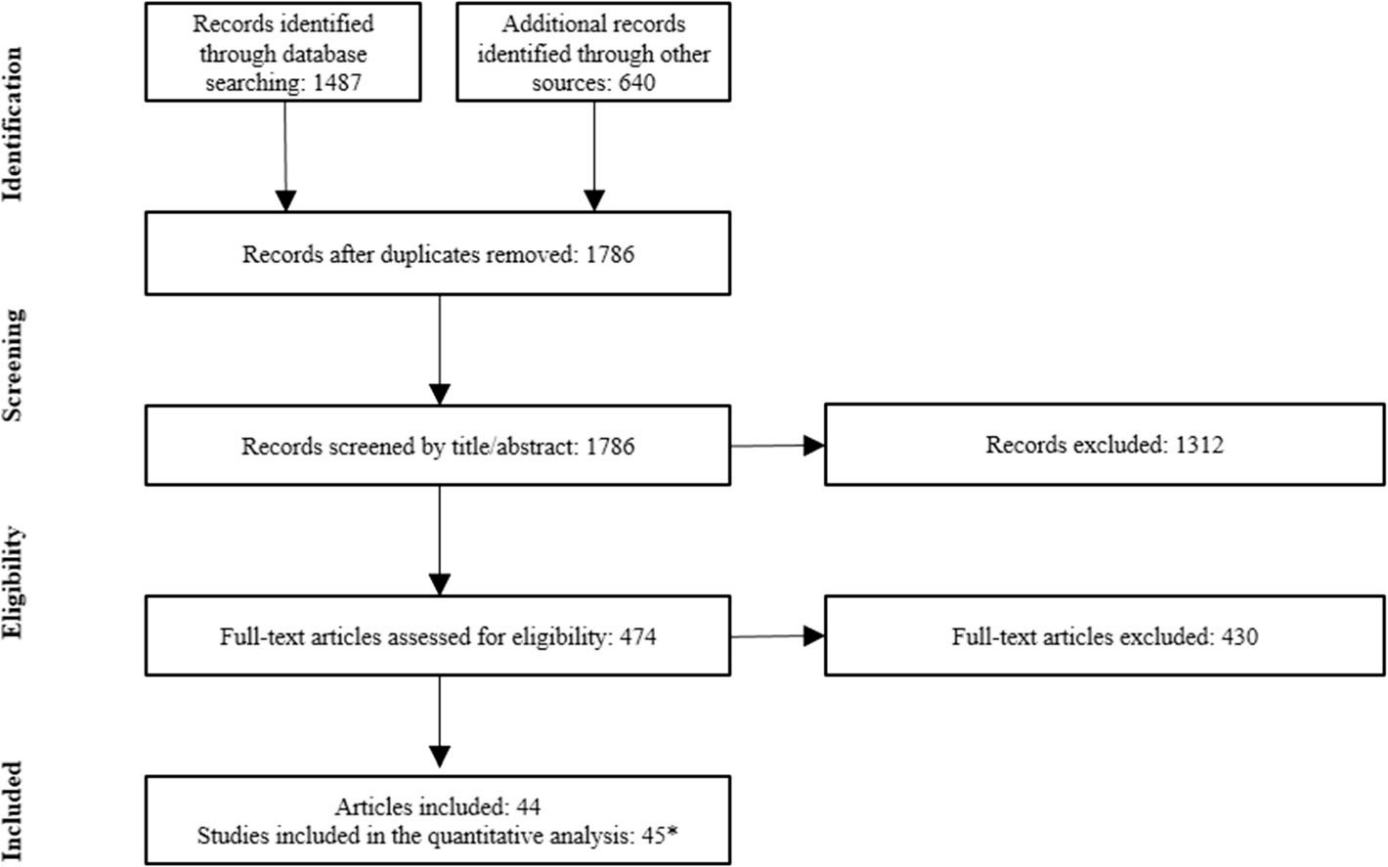
PRISMA flow diagram of study selection

One thousand seven hundred and eighty-six unique records were identified through the search strategy and the similarity search. Four hundred and seventy-four records remained after the title and abstract screening and 44 publications remained after the full-text screening. The 44 publications reported data from 45 RCTs and 170,794 participants (85,544 participants in the ARB group and 85,250 participants in the placebo/control group) (Fig. 1). The publication by Chaturvedi et al. [15] reported findings from two RCTs, the DIRECT-Prevent 1 and DIRECT-Protect 1 studies.

Twenty four RCTs compared ARBs versus placebo, while 21 RCTs against an active medication. The majority of RCTs (*n* = 39) included a larger proportion of males (ranging from 54 to 90%). Only two RCTs, DIRECT-Prevent 1 and DIRECT-Protect 1 enrolled participants with a median age < 50 years. Among the studies that reported the median BMI, only 22% had participants with a normal BMI (< 25 kg/cm^2^). Fourteen, nine, and eight RCTs included only patients with hypertension, chronic heart failure, and diabetes mellitus, respectively (Table 1). All-cause mortality, myocardial infarction, and stroke were assessed in 39, 37, and 36 RCTs.

**Table 1:** Characteristics of the RCTs included in the meta-analysis

Trial name, year publication	Population	Setting	Intervention	Control	Follow up (in months)	Male (%)	Mean / median age (years)	Mean BMI (kg/m^2^)	Mean cholesterol (mg/dL)	Mean LDL (mg/dL)	Mean HDL (mg/dL)	Mean triglyceride (mg/dL)	Non-smoker (%)	Hypertension (%)	Heart failure (%)	Diabetes mellitus (%)	Ischaemic / coronary artery disease (%)	Chronic kidney disease (%)
4C (2016) [16]	Patients with IHD after coronary stent implantation	39 centres in Japan	Candesartan	Standard care without ARB	36	73	69	24	NR	111	49	140	83	73	8	35	100	NR
ACTIVE I (2011) [17]	Patients with atrial fibrillation	600 centres worldwide	Irbesartan	Placebo	54	61	70	29	NR	NR	NR	NR	50	88	32	20	NR	NR
CARP (2011) [18]	Patients that received a coronary stent	5 centres in Hiroshima, Japan	Valsartan	Non-ARB therapy	48	79	65	24	NR	NR	NR	NR	50	75	NR	43	100	30
CASE-J (2008) [19]	Patients with high-risk hypertension	527 physicians from Japan	Candesartan	Amlodipine	41	55	64	25	NR	NR	NR	NR	79	100	0	43	43	24
CHARM-Added (2003) [20]	Patients with CHF and LVEF< 40	618 centres in 26 countries	Candesartan	Placebo	41	79	64	28	NR	NR	NR	NR	83	48	100	30	68	NR
CHARM-Alternative (2003) [7]	Patients with symptomatic CHF and LVEF< 40%	618 centres in 26 countries	Candesartan	Placebo	34	68	67	28	NR	NR	NR	NR	86	50	100	27	62	NR
CHARM-Preserved (2003) [21]	Patients with HF and LVEF> 40	618 centres in 26 countries	Candesartan	Placebo	37	60	67	29	NR	NR	NR	NR	87	64	100	28	56	NR
Cice et al. (2010) [22]	Patients with CHF and in haemodialysis	30 clinics in Italy	Telmisartan	Placebo	36	90	63	NR	NR	NR	NR	NR	61	NR	100	29	57	100
DETAIL (2004) [23]	Patients with diabetes mellitus and nephropathy	39 centres in northern Europe	Telmisartan	Enalapril	60	73	61	31	223	137	48	207	37	100	0	100	NR	100
DIRECT-Prevent 1 (2008) [15]	Patients with type 1 diabetes a no retinopathy	309 centres worldwide	Candesartan	Placebo	56	56	30	24	184	NR	66	NR	74	NR	NR	100	NR	0
DIRECT-Protect 1 (2008) [15]	Patients with type 1 diabetes and retinopathy	309 centres worldwide	Candesartan	Placebo	56	57	32	25	186	NR	66	NR	74	NR	NR	100	NR	0
DIRECT-Protect 2 (2008) [24]	Patients with type 2 diabetes and retinopathy	309 centres worldwide	Candesartan	Placebo	56	50	57	29	205	NR	NR	NR	73	62	NR	100	5	0
E-COST (2005) [25]	Patients with hypertension	Centres in Saitama, Japan	Candesartan	Non-ARB therapy	37	48	NR	NR	NR	NR	NR	NR	NR	100	0	0	0	NR
E-COST-R (2005) [26]	Patients with hypertension and mild renal impairment	Centres in Saitama, Japan	Candesartan	Non-ARB therapy	37	59	67	NR	181	NR	NR	NR	NR	100	0	0	6	100
ELITE (1997) [27]	Patients with CHF and LVEF< 40%	125 centres in the USA, Europe, and South America	Losartan	Captopril	13	67	74	NR	NR	NR	NR	NR	88	57	100	25	50	7
ELITE II (2000) [28]	Patients with CHF and LVEF< 40%	289 centres in 46 countries	Losartan	Captopril	23	69	71	NR	NR	NR	NR	NR	NR	49	100	24	79	NR
GISSI-AF (2009) [29]	Patients with history of atrial fibrillation	100 centres in Italy	Valsartan	Placebo	12	62	68	28	NR	NR	NR	NR	81	85	8	15	12	3
HIJ-CREATE (2009) [30]	Patients with coronary artery disease and hypertension	14 centres in Japan	Candesartan	Non-ARB therapy	50	80	66	25	193	NR	45	128	64	100	21	38	100	NR
HOPE-3 (2016) [31]	Patients with intermediate cardiovascular risk	228 centres in 21 countries	Candesartan + hydrochlorothiazide	Placebo	67	54	66	27	201	128	45	128	72	38	0	5.8	0	0
IDNT (2003) [32]	Patients with diabetes mellitus and nephropathy	Centres in the North America, Europe, Latin America, South East Asia, and Oceania	Irbesartan	Amlodipine or placebo^a^	31	64	59	31	NR	NR	NR	NR	NR	100	0	100	28	100
I-PRESERVE (2008) [33]	Patients with CHF and LVEF > 45%	Centres in 25 countries	Irbesartan	Placebo	50	40	72	30	NR	NR	NR	NR	NR	89	100	28	0	0
IRMA-2 (2001) [34]	Patients with hypertension, diabetes mellitus, and micro-albuminuria	96 centres worldwide	Irbesartan^b^	Placebo	24	69	58	30	224	140	44	180	81	100	NR	100	6	0
J-RHYTHM II (2011) [35]	Patients with hypertension and atrial fibrillation	48 centres in Japan	Candesartan	Amlodipine	12	69	66	NR	NR	NR	NR	NR	NR	100	3	9	1	NR
Kondo et al. (2003) [36]	Patients with history of coronary intervention	Ogaki Municipal Hospital in Japan	Standard care + Candesartan	Standard care without candesartan	24	76	65	24	187	114	49	126	76	44	2	25	100	NR
KYOTO HEART (2009) [37]	Patients with uncontrolled hypertension	31 centres from Kyoto, Japan	Valsartan	Non-ARB therapy	39	57	66	39	NR	122	55	149	78	100	7	27	23	NR
LIFE (2002) [38]	Patients with hypertension and left ventricular hypertrophy	830 centres from the USA, the UK, and Scandinavia	Losartan	Atenolol	58	46	67	28	232	NR	58	NR	84	100	0	13	16	NR
MOSES (2005) [39]	High-risk hypertensive patients	Centres in Germany and Austria	Eprosartan	Nitredipine	45	54	68	28	NR	NR	NR	NR	NR	100	26	37	26	5.4
NAVIGATOR (2010) [40]	Patients with impaired glucose tolerance	806 centres in 40 countries	Valsartan	Placebo	60	49	64	31	210	127	50	151	89	78	NR	49	12	11
OCTOPUS (2013) [41]	Patients with hypertension and in haemodialysis	66 dialysis centres in Okinawa, Japan	Olmesartan	Non-ARB therapy	60	62	60	24	155	NR	NR	155	65	100	NR	32	7	100
ONTARGET (2008) [42]	Patients with coronary, peripheral, cerebrovascular disease or diabetes with end-organ damage	733 centres in 40 countries	Telmisartan	Ramipril or ramipril + telmisartan^c^	56	77	66	28	190	112	50	151	36	69	0	37	75	NR
OPTIMAAL (2002) [43]	Patients with acute myocardial infarction and heart failure	329 centres in 7 European countries	Losartan	Captopril	35	69	67	27	212	130	45	168	NR	36	6	17	100	NR
ORIENT (2011) [44]	Patients with diabetes mellitus with proteinuria	Centres in Japan and Hong Kong	Olmesartan	Placebo	38	69	59	25	208	NR	NR	NR	75	100	4	100	5	100
PRoFESS (2008) [45]	Patients with a recent ischaemic stroke	695 centres in 35 countries	Telmisartan	Placebo	30	64	66	27	NR	NR	NR	NR	43	74	3	28	NR	NR
RENAAL (2001) [46]	Patients with diabetes and nephropathy	250 centres in 28 countries	Losartan	Placebo	41	63	60	30	228	142	45	219	82	93	0	100	11	100
ROAD (2007) [47]	Patients with proteinuria and chronic renal insufficiency	Nanfang Hospital Renal Division in China	Losartan	Benazepril	44	63	50	23	97	NR	NR	177	NR	63	0	0	0	100
SCAST (2011) [48]	Patients with acute stroke	146 centres in Europe	Candesartan	Placebo	6	58	71	NR	NR	NR	NR	NR	NR	70	NR	16	NR	NR
SCOPE (2003) [49]	Patients with mild to moderate elevated blood pressure	527 centres in Europe	Candesartan	Placebo	45	36	76	27	239	NR	NR	NR	91	52	NR	12	4	NR
SUPPORT (2015) [50]	Patients with hypertension and CHF	17 centres in Tohoku, Japan	Olmesartan	Non-ARB therapy	53	75	66	25	NR	108	NR	NR	NR	100	100	50	47	0
Suzuki et al. (2008) [51]	Patients with kidney failure treated with haemodialysis	5 dialysis centres in Saitama, Japan	Losartan, candesartan, or valsartan	Non-ARB therapy	36	59	60	21	157	NR	NR	NR	78	93	16	52	2	100
Takahashi et al. (2006) [52]	Patients with kidney failure treated with haemodialysis	Enshu General Hospital in Japan	Candesartan	Nothing	19	58	61	20	NR	NR	NR	NR	NR	81	0	33	0	100
TRANSCEND (2008) [53]	Patients with coronary, peripheral, cerebrovascular disease or diabetes with end-organ damage, and intolerant to ACE inhibitors	630 centres in 40 countries	Telmisartan	Placebo	56	57	67	28	197	117	49	158	47	76	0	36	74	NR
T-VENTURE (2009) [54]	Patients with acute myocardial infarction	4 centres in Japan	Valsartan	ACE inhibitor therapy	6	83	63	NR	NR	NR	NR	NR	40	57	0	34	100	NR
Val-HeFT (2001) [55]	Patients with heart failure	302 centres in 16 countries	Valsartan	Placebo	23	80	63	NR	NR	NR	NR	NR	NR	NR	100	25	57	NR
VALIANT (2003) [56]	Patients with recent myocardial infarction and LVEF < 35%	931 centres in 24 countries	Valsartan	Captopril^d^	25	78	65	27	NR	NR	NR	NR	NR	56	15	23	100	NR
VALUE (2004) [6]	Patients with hypertension and high risk of cardiac event	Centres in 31 countries	Valsartan	Amlodipine	50	58	67	29	NR	NR	NR	NR	NR	93	6	NR	45	NR

*ACE* Angiotensin-converting enzyme, *ARB* Angiotensin II receptor blockers, *CHF* Congestive heart failure, *IHD* Ischaemic heart disease, *LVEF* Left-ventricular ejection fraction, *NR* Not reported

^a^IDNT (2003): Two control groups, placebo group was excluded

^b^IRMA-2 (2001): Two intervention groups, irbesartan 150 mg daily and irbesartan 300 mg daily were combined

^c^ONTARGET (2008): Three intervention groups, ramipril + telmisartan group was excluded

^d^VALIANT (2003) Three intervention groups, valsartan + captopril group was excluded

### Quantitative synthesis

**Fig. 2:**
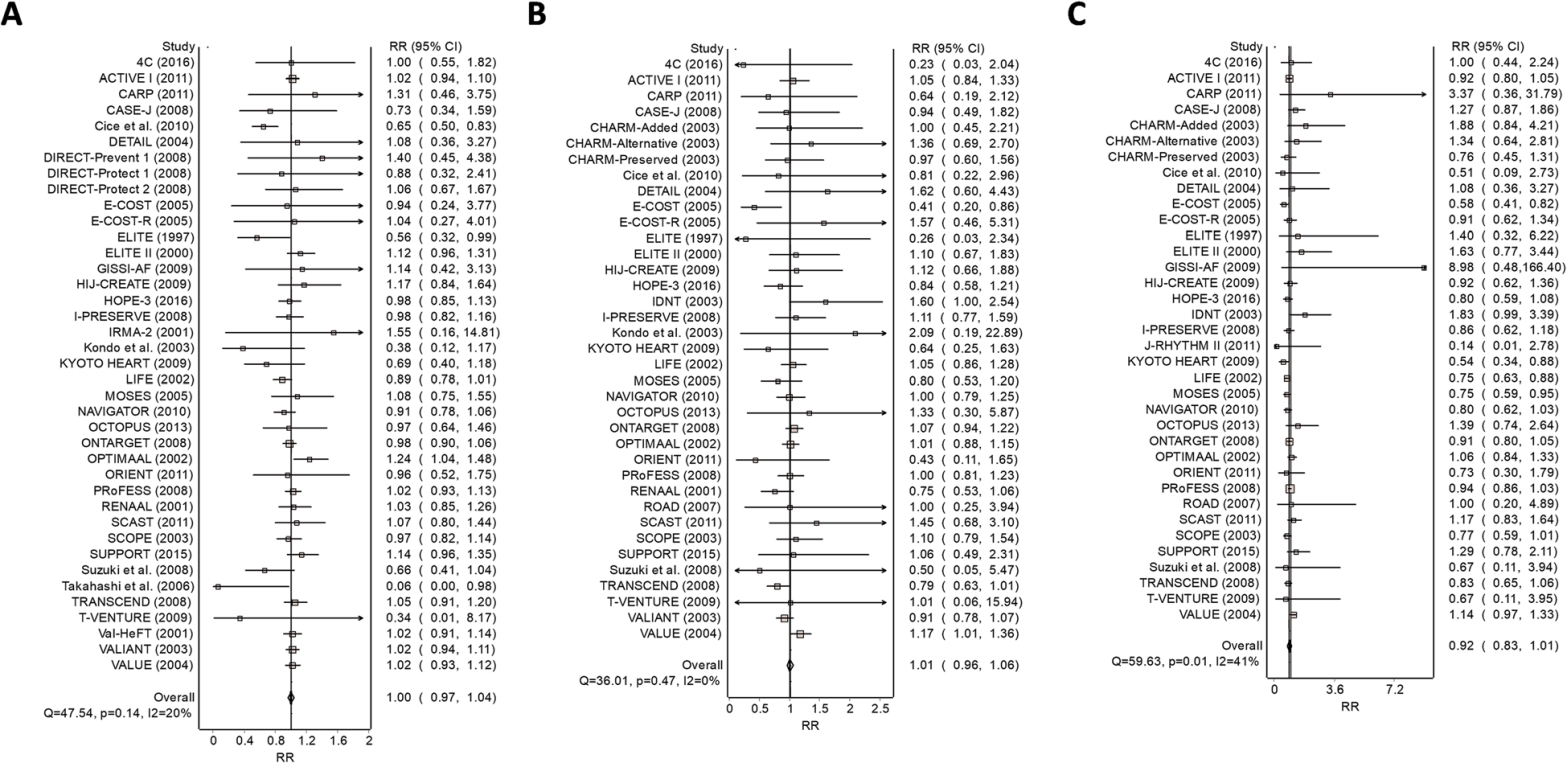
Forest plot depicting the relative risk of ARBs on a) all-cause mortality, b) myocardial infarction, and c) stroke

After pooling all the available evidence, it was found that ARBs do not increase the risk of all-cause mortality (RR 1.00; 95%CI 0.97–1.04), myocardial infarction (RR 1.01; 95%CI 0.96–1.06), or stroke (RR 0.92; 95%CI 0.83–1.01) (Fig. 2). Sensitivity analyses based on different study and participants characteristics showed no increase in risk of any of the three outcomes of interest. However, it was also noticed that ARBs did not reduce the risk of all-cause mortality (RR 0.99; 95%CI 0.95–1.04) or myocardial infarction (RR 0.96; 95%CI 0.88–1.05) when compared to placebo, ARBs only decreased the risk of stroke (RR 0.91; 95%CI 0.85–0.98) (Table 2). Sensitivity analyses also revealed a decreased in all-cause mortality risk with ARBs when the proportion of smokers is small (< 25%) (RR 0.91; 95%CI 0.84–0.98); and stroke in females (RR 0.76; 95%CI 0.68–0.84), patients with elevated total cholesterol (RR 0.82; 95%CI 0.82–0.91) and lower levels of HDL (RR 0.90; 95%CI 0.80–0.98) (Table 2).

**Table 2:** Sensitivity analyses

	All-cause mortality			Myocardial infarction			Stroke		
	RR (95%CI)	*I*^2^	N	RR (95%CI)	*I*^2^	N	RR (95%CI)	*I*^2^	N
Type of control									
Placebo	0.99 (0.95–1.04)	13	18	0.96 (0.88–1.05)	0	14	**0.91 (0.85–0.98)**	7	14
Active	1.01 (0.95–1.08)	28	21	1.03 (0.96–1.11)	7	23	0.93 (0.79–1.08)	54	22
Active only ACE inhibitors	1.04 (0.95–1.13)	46	8	1.01 (0.93–1.09)	0	9	0.98 (0.88–1.10)	0	8
Follow-up period									
≤ 40 weeks	1.01 (0.91–1.14)	51	19	0.98 (0.88–1.10)	12	18	0.94 (0.74–1.20)	40	18
> 40 weeks	1.00 (0.96–1.03)	0	20	1.03 (0.96–1.10)	0	19	0.90 (0.82–1.00)	45	18
Proportion of males									
≤ 50%	0.93 (0.86–1.00)	0	6	1.02 (0.85–1.22)	37	5	**0.76 (0.68–0.84)**	0	5
> 50%	1.02 (0.97–1.06)	23	33	1.01 (0.95–1.07)	0	32	0.96 (0.87–1.05)	28	31
Age									
≤ 65 years	0.98 (0.88–1.09)	32	18	0.95 (0.85–1.06)	0	15	1.03 (0.80–1.34)	22	12
> 65 years	1.01 (0.98–1.05)	10	20	1.04 (0.98–1.10)	0	21	0.92 (0.84–1.00)	41	23
BMI									
Normal range	0.84 (0.60–1.19)	31	7	0.81 (0.41–1.57)	0	6	1.21 (0.77–1.90)	0	5
Overweight and obese	1.01 (0.98–1.04)	0	24	1.01 (0.96–1.07)	5	24	0.92 (0.83–1.01)	49	23
Elevated total cholesterol									
≥ 200 mg/dL	0.98 (0.91–1.05)	15	10	0.99 (0.91–1.08)	0	8	**0.82 (0.74–0.91)**	6	7
Elevated LDL									
≥ 120 mg/dL	1.01 (0.90–1.14)	36	7	0.97 (0.87–1.07)	0	6	0.86 (0.70–1.07)	45	5
Decreased HDL									
< 50 mg/dL	1.01 (0.95–1.08)	15	11	0.99 (0.89–1.09)	20	10	**0.90 (0.82–0.98)**	0	8
Elevated triglyceride									
≥ 150 mg/dL	1.01 (0.94–1.08)	13	8	0.99 (0.90–1.09)	16	8	0.92 (0.83–1.01)	0	7
Proportion of smokers									
< 25%	**0.91 (0.84–0.98)**	2	12	0.99 (0.88–1.11)	0	13	0.81 (0.67–0.99)	41	12
≥ 25%	0.99 (0.95–1.05)	7	15	0.99 (0.91–1.01)	0	12	0.92 (0.87–0.98)	0	12
Hypertension									
Only patients with hypertension	0.98 (0.89–1.07)	0	12	1.02 (0.80–1.29)	27	12	0.82 (0.66–1.03)	57	13
Chronic heart failure (CHF)									
Only patients without CHF	0.97 (0.92–1.03)	0	11	0.99 (0.83–1.18)	43	12	0.85 (0.73–1.00)	47	11
Only patients with CHF	1.00 (0.85–1.19)	75	6	1.06 (0.86–1.32)	0	8	1.04 (0.81–1.32)	14	8
Diabetes mellitus (DM)									
Only patients without DM	0.99 (0.38–2.61)	0	2	0.65 (0.26–1.59)	48	3	0.72 (0.50–1.04)	37	3
Only patients with DM	1.04 (0.88–1.23)	0	7	0.99 (0.53–1.80)	67	4	1.31 (0.73–2.35)	30	3
Ischemic/coronary artery disease									
Only patients with ischemic/coronary artery disease	1.06 (0.91–1.22)	25	7	0.97 (0.88–1.07)	0	7	1.02 (0.84–1.24)	0	5
Chronic kidney disease									
Only patients with chronic kidney disease	0.86 (0.66–1.12)	50	8	0.99 (0.71–1.41)	20	9	1.08 (0.83–1.39)	0	8

*CI* confidence interval; *N* number of studies; *RR* relative risk; *ACE* angiotensin-converting-enzyme

Statistically significant results are emboldened

The most common deficiencies were no blinding of participants and personnel (*n* = 14; 31%), followed by no blinding of the outcome assessor (*n* = 10; 22%) and incomplete outcome data (*n* = 10; 22%). Overall, the RCTs showed low risk of bias except for E-COST [25], E-COST-R [26], and Kondo et al. [36] (S2).

The Doi plots revealed minor asymmetry for all-cause mortality (*LFK* index = − 1.24) and myocardial infarction (*LFK* index = − 1.33) for RCTs reporting favourable results for ARBs. No asymmetry was observed for stroke (supplementary material S3).

## Discussion

Findings from previous RCTs were controversial, the VALUE [6] and the CHARM-alternative [7] trials found increase in myocardial infarction with ARBs compared to amlodipine and placebo, respectively. While other large RCTs such as the LIFE [38] and the RENAAL [46] trials found a decrease in all-cause of death and myocardial infarction with ARBs. In 2011, Bangalore et al. [57] conducted a meta-analysis on ARBs and the risk of myocardial infarction and found that ARBs do not increase the risk of cardiovascular events. Since then, multiple RCTs have been published; in our meta-analysis we pooled the most updated evidence (45 RCTs comprising of 170,794 participants – 8 RCTs and 23,000 more participants that Bangalore et al.) and corroborated that ARBs are safe medications as they do not increase the risk of all-cause mortality, myocardial infarction, or stroke. It is worth pointing out that our meta-analysis (in line with previous studies [57, 58]) also found that ARBs do not reduce the risk of all-cause mortality and myocardial infarction when compared to placebo.

In addition, the safety profile of ARBs was examined in multiple scenarios by restricting the analysis to different study and participants characteristics (i.e. sensitivity analyses). In none of the cases, ARBs were found to increase the risk of all-cause mortality, myocardial infarction, and stroke. ARBs reduce the risk of all-cause mortality by 9% in populations with low prevalence of smokers and exerts a cerebrovascular protective effect in female patients and patients with abnormal total cholesterol or HDL.

Findings from our study are reassuring for patients and clinicians as ARBs are widely used to treat conditions such as hypertension, chronic kidney disease/kidney failure (especially in patients with diabetes mellitus), and heart failure. However, the findings need to be understood in light of some of the limitations. Only RCTs were included, but the possibility of confounding not accounted during the analysis of the RCTs cannot be completely ruled out. There was heterogeneity in the RCTs protocols (e.g. inclusion criteria, different ARBs, different doses, follow-up) that needs to be accounted in future research synthesis studies through individual patients meta-analysis.

## Conclusion

In conclusion, our meta-analysis provides reassuring evidence for patients and clinicians that ARBs are safe drugs, and do not increase the risk of death, myocardial infarction, and stroke.

## Supplementary information

**Additional file 1: S1.** Search strategy **S2.** Risk of bias of the included studies **S3.** Doi (top) and funnel (bottom) plots for the studies assessing a) all-cause mortality, b) myocardial infarction, and c) stroke **S4**.

## Data Availability

he data used in the study was extracted from published studies.

## Abbreviations

ARB: Angiotensin receptor blocker
ACE: Angiotensin-converting-enzyme
CVD: Cardiovascular disease
DALYs: Disability adjusted life years
IVhet: Inverse variance heterogeneity
RCT: Randomised controlled trial
RR: Relative risk
