# Supplementary material for: Assessing the risk of angiotensin receptor blockers on major cardiovascular events: a systematic review and meta-analysis of randomized controlled trials
Source: BMC Cardiovasc Disord. 2020 Apr 21;20:188. doi: 10.1186/s12872-020-01466-5 (PMC7175553; doi:10.1186/s12872-020-01466-5)
Supplement: Supplementary file 1 — Additional file 1: S1. Search strategy S2. Risk of bias of the included studies S3. Doi (top) and funnel (bottom) plots for the studies assessing a) all-cause mortality, b) myocardial infarction, and c) stroke S4. [file 12872_2020_1466_MOESM1_ESM.docx]

**Supplementary material**

**S1.** Search strategy

(("randomized controlled trial"[Publication Type] OR "randomized controlled trials as topic"[MeSH Terms] OR "randomized controlled trial"[All Fields] OR "randomised controlled trial"[All Fields])

AND

("angiotensin receptor antagonists"[Pharmacological Action] OR "angiotensin receptor antagonists"[MeSH Terms] OR ("angiotensin"[All Fields] AND "receptor"[All Fields] AND "antagonists"[All Fields]) OR "angiotensin receptor antagonists"[All Fields])

AND

(("hospital mortality"[MeSH Terms] OR ("hospital"[All Fields] AND "mortality"[All Fields]) OR "hospital mortality"[All Fields]) OR ("Treatment Outcome"[Mesh]))

AND

("cardiovascular diseases"[MeSH Terms] OR ("cardiovascular"[All Fields] AND "diseases"[All Fields]) OR "cardiovascular diseases"[All Fields] OR ("cardiovascular"[All Fields] AND "disease"[All Fields]) OR "cardiovascular disease"[All Fields]))

**S2.** Risk of bias of the included studies

| Trial name, year publication | Random sequence generator | Allocation concealment | Selective reporting  endpoint | Blinding of participants and personnel | Blinding of outcome assessment | Incomplete outcome data  (follow up) |
| --- | --- | --- | --- | --- | --- | --- |
| 4C (2016) | Low | Unclear | Low | High | Low | Low |
| ACTIVE I (2011) | Low | Low | Low | Low | Low | High |
| CASE-J (2008) | Low | Low | Low | High | Low | Low |
| CARP (2011) | Low | Unclear | Unclear | High | Unclear | Low |
| CHARM-Alternative (2003) | Low | Unclear | Low | Low | Low | Low |
| CHARM-Added (2003) | Low | Unclear | Low | Low | Low | Low |
| CHARM-Preserved (2003) | Low | Unclear | Low | Low | Low | Low |
| Cice et al. (2010) | Low | Unclear | Low | Low | Unclear | High |
| DETAIL (2004) | Low | Low | Low | Low | Unclear | High |
| DIRECT-Prevent 1 (2008) | Low | Low | Low | Low | Low | Low |
| DIRECT-Protect 1 (2008) | Low | Low | Low | Low | Low | Low |
| DIRECT-Protect 2 (2008) | Low | Low | Low | Low | Low | Low |
| E-COST (2005) | Low | Unclear | Low | High | High | High |
| E-COST-R (2005) | Low | Unclear | Low | High | High | Unclear |
| ELITE (1997) | Low | Unclear | Low | Low | Unclear | High |
| ELITE II (2000) | Low | Unclear | Low | Low | Unclear | Low |
| GISSI-AF (2009) | Low | Low | Low | Low | Low | Low |
| HIJ-CREATE (2009) | Low | Low | Low | High | Low | Low |
| HOPE-3 (2016) | Low | High | Low | Low | High | Low |
| IDNT (2003) | Low | Low | Low | Low | Low | Low |
| I-PRESERVE (2008) | Low | Unclear | Low | Low | Unclear | High |
| IRMA-2 (2001) | Low | Low | Low | Low | Unclear | Low |
| J-RHYTHM II (2011) | Low | Unclear | Low | High | High | Low |
| Kondo et al. (2003) | Low | Unclear | Unclear | High | High | Low |
| KYOTO HEART (2009) | Low | Low | Low | High | Low | Low |
| LIFE (2002) | Low | Unclear | Low | Low | High | High |
| MOSES (2005) | Low | Unclear | Low | Unclear | Low | Low |
| NAVIGATOR (2010) | Low | Low | Low | Low | Low | High |
| OCTOPUS (2013) | High | Unclear | Low | High | Low | Low |
| ONTARGET (2008) | Low | Unclear | Low | Low | Low | High |
| OPTIMAAL (2002) | Low | Low | Low | Low | Low | High |
| ORIENT (2011) | Low | Low | Low | Low | Low | Unclear |
| PRoFESS (2008) | Low | Unclear | Low | Low | Unclear | Low |
| RENAAL (2001) | Low | Low | Low | Low | Low | Unclear |
| ROAD (2007) | Low | High | Low | High | Low | Low |
| SCAST (2011) | Low | Unclear | Low | Low | Unclear | Low |
| SCOPE (2003) | Unclear | High | Low | Low | High | Low |
| SUPPORT (2015) | Unclear | Unclear | Low | Low | High | Low |
| Suzuki et al. (2008) | Low | Low | Unclear | High | High | Low |
| Takahashi et al. (2006) | Low | Low | Unclear | High | High | Low |
| TRANSCEND (2008) | Low | Low | Low | High | Low | Low |
| T-VENTURE (2009) | Low | Unclear | Unclear | Unclear | Unclear | Low |
| Val-HeFT (2001) | Low | Low | Low | Low | Low | Low |
| VALIANT (2003) | Low | Low | Low | Low | Low | Low |
| VALUE (2004) | Low | Unclear | Low | Low | Low | Low |
|  | | | | | | |

| **A**  **** | **B**  **** | **C**  **** |
| --- | --- | --- |
|  |  |  |
| **S3.** Doi (top) and funnel (bottom) plots for the studies assessing a) all-cause mortality, b) myocardial infarction, and c) stroke | | |

|  |  |  |
| --- | --- | --- |
| **S4.** | | |
